# Ultrafine Aerosol Particle Sizer Based on Piezoresistive Microcantilever Resonators with Integrated Air-Flow Channel

**DOI:** 10.3390/s21113731

**Published:** 2021-05-27

**Authors:** Maik Bertke, Ina Kirsch, Erik Uhde, Erwin Peiner

**Affiliations:** 1 Institute for Semiconductor Technology and Laboratory for Emerging Nanometrology (LENA), Technische Universität Braunschweig, Hans-Sommer-Str. 66/Langer Kamp 6a, 38106 Braunschweig, Germany; m.bertke@tu-bs.de; 2 Fraunhofer Wilhelm-Klauditz-Institut (WKI), Bienroder Weg 54E, 38106 Braunschweig, Germany; ina.kirsch@wki.fraunhofer.de (I.K.); erik.uhde@wki.fraunhofer.de (E.U.)

**Keywords:** differential mobility particle sizer, micro electro mechanical system, micro-fluidic channel, piezoresistive micro cantilever, picogram balance, ultrafine particles, carbon aerosol

## Abstract

To monitor airborne nano-sized particles (NPs), a single-chip differential mobility particle sizer (DMPS) based on resonant micro cantilevers in defined micro-fluidic channels (µFCs) is introduced. A size bin of the positive-charged fraction of particles herein is separated from the air stream by aligning their trajectories onto the cantilever under the action of a perpendicular electrostatic field of variable strength. We use previously described µFCs and piezoresistive micro cantilevers (PMCs) of 16 ng mass fabricated using micro electro mechanical system (MEMS) technology, which offer a limit of detection of captured particle mass of 0.26 pg and a minimum detectable particulate mass concentration in air of 0.75 µg/m^3^. Mobility sizing in 4 bins of a nebulized carbon aerosol NPs is demonstrated based on finite element modelling (FEM) combined with a-priori knowledge of particle charge state. Good agreement of better than 14% of mass concentration is observed in a chamber test for the novel MEMS-DMPS vs. a simultaneously operated standard fast mobility particle sizer (FMPS) as reference instrument. Refreshing of polluted cantilevers is feasible without de-mounting the sensor chip from its package by multiply purging them alternately in acetone steam and clean air.

## 1. Introduction

Air quality is a most important factor of a healthy life with high current relevance. For example, long-term exposure to a polluted ambient can be a similar hazard to life expectancy as inhaling tobacco smoke and about 15% of the worldwide deaths from the current coronavirus disease 2019 (COVID-19) pandemic might be related to a long-lasting exposure to air polluted by anthropogenic sources (e.g., particulate matter (PM) from combustion sources) [1,2]. Air-polluting PM is categorized according to its size, i.e., the commonly monitored PM_10_, PM_2.5_ and PM_1_ fractions denoting PM of aerodynamic diameter less than 10 µm, 2.5 µm and 1 µm, respectively. Moreover, ultrafine particles (UFPs) or PM_0.1_ of a diameter of less than 100 nm have been recognized, which are in large amounts generated by traffic, e.g., through combustion by diesel (20 to 130 nm) and gasoline engines (40 to 80 nm) [3]. In air, PM_0.1_ is more persistent than larger particulates and can penetrate lungs deeper with the ability to translocate into the circulatory system. Oxidative stress associated with exposure to UFPs comprising hazardous components can cause inflammatory processes, which can lead to significant consequences to the respiratory conditions as well as genotoxic, mutagenic, and carcinogenic activity in human beings depending on the additives’ toxicity [3]. For example, cerium dioxide and titanium dioxide instilled on lung epithelial cells and macrophages can either trigger moderate effects or initiate cytotoxicity, pro-inflammatory gene expression and genotoxicity [4]. On blood cell surfaces UFPs may activate angiotensin-converting enzyme 2 (ACE-2) receptors, which are known to be involved in the infection process of cells by COVID-19 [2]. 

A most practicable way to protect people from excessive exposure to UFP pollution is to provide them with lightweight and battery-powered personal monitors. For indoor personal exposure monitoring, low-cost optical particle counters based on light scattering are commercially available, which, however, cannot accurately measure particles smaller than 0.3 µm [3,5,6]. A minimum particle size of 140 nm was reported for a Fresnel ring lens-based optical particle counter, while counters based on complementary metal-oxide-semiconductor (CMOS) image sensing or holographic detection combined with extensive image processing are limited to the particle size range above 3 µm [7]. 

Gravimetric methods using resonant sensors, e.g., based on micro or nano electro mechanical systems (M/NEMS) can provide a sufficiently high sensitivity to detect nano-sized particles (NPs) [8]. For example, TiO_2_ NPs were sampled on an array of tiny Si nanowire resonators and detected by a down-shift of resonance frequency in a scanning electron microscope [9]. The reported mass resolution of 31.6 fg corresponds to limit of detection (LOD) of 5 to 6 NPs of a mean diameter of 125 nm. A direct mass-reading device was demonstrated by a weakly coupled piezoelectric MEMS-resonator sensor showing an LOD of 2.6 pg with 100 nm soot particles [10]. Unfortunately, particle size distributions have not been measured so far using such M/NEMS resonant sensors, which would nevertheless be necessary for a more comprehensive evaluation of air quality. 

Owing to their sensitive dynamic response, micro cantilevers have attracted considerable interest for high-resolution mass sensing [11]. Correspondingly, we report in this study on a piezoresistive micro cantilever (PMC) for NP size distribution measurement, which is integrated in a micro-fluidic channel (µFC). Based on mobility separation of charged particles flowing in the µFC, we describe a procedure for analyzing the size distribution of polydisperse aerosols at different sampling voltages, i.e., the MEMS device is operated as a differential mobility particle sizer (MEMS-DMPS). For validating this concept we use carbon engineered NPs, which can be considered as a model aerosol for environmental PM_0.1_, whose mass is largely composed of carbonaceous material (elemental, organic or black carbon). 

In the following paper, we first briefly review and evaluate in Section 2 a selection of recently reported portable gravimetric airborne particle sensors [12,13,14,15,16,17,18,19,20,21,22,23,24,25]. In Section 3, we describe design, fabrication and test of a recently proposed highly sensitive MEMS-based NP sensor containing a µFC with an integrated PMC [26,27]. As the essential outcome of the present work, a novel procedure is then described in Section 4 for retrieving particle-size distributions of a carbon NP aerosol from the sensor output. Finally, regeneration of a NP-loaded cantilever is addressed in Section 5.

## 2. Portable Gravimetric Aerosol Monitors with Particle-Size Separation

Size-selective gravimetric PM sensing is based on particle sampling on a resonant device using aerodynamic (filter, impactor), electrostatic (mobility analyzer), or thermophoretic techniques. Numerous sampling-and-separation techniques have been reported for direct-reading/real-time PM monitoring based on micro filter (µF), impactor filter (IF), virtual impactor (VI), cascade impactor (CI), differential mobility analyzer (DMA), etc. [12]. A selection of recently published wearable devices and their characteristics is presented in Table 1. Such gravimetric sensors can monitor PM_1_ and PM_2.5_ of concentrations as low as 0.7 µg/m^3^ to 50 µg/m^3^ and are based on MEMS electrothermal-piezoresistive cantilever (MEMS-EPC), thermal-piezoresistive resonator/oscillator MEMS-TPR/O, on micro quartz tuning fork (MQTF), surface acoustic wave resonator (SAW), or film bulk acoustic resonator (FBAR) devices. Particle-size separation is realized by filters and/or impactors, which remove particles from the air flow above a certain size limit set by the particle range to be captured on the resonator.

**Figure 1:**
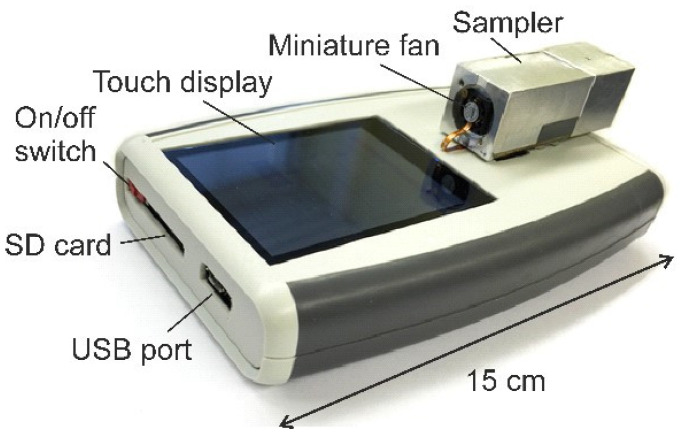
Pocket-size, battery-powered NP monitor “Cantor” with a weight of 0.4 kg and a power consumption of 1.25 W.

The portable (0.4 kg) and battery-powered (1.25 W) particle monitor Cantor (Figure 1) has a micro filter (µF) or an impactor filter (IF) at its inlet for separating coarse particles of 2.5 µm and 1 µm, respectively, from a sucked-in air flow and was shown to sample carbon, titanium, silica and silver NPs on an electrothermal-piezoresistive cantilever (MEMS-EPC) [13,25]. Recently, its limit of detection of LOD = 3 × *δf*/*S* = 15 µg/m^3^ (with the minimum detectable frequency shift rate *δf*/*δt* and the mass-concentration sensitivity *S*) could be improved to 1.4 µg/m^3^ by reducing a parasitic feedthrough between the thermal actuator and the piezoresistive Wheatstone bridge [13]. In this report, furthermore, using a tiny commercial piezoresistive atomic-force-microscopy cantilever (mass of ~31 µg, i.e., by a factor 20 lower than the Cantor cantilever) with thermal bimorph actuator, the LOD was further reduced to 0.7 µg/m^3^. 

For micro-particle separation and detection, a µFC with micro filter (µF) was proposed for collecting particles larger than the filter spacing of 1.1 µm [15]. The µFC was integrated in a MEMS-EPC for resonant gravimetric detection. Alternatively, a gated CMOS thermal-piezoresistive oscillator (MEMS-TPO) with an impactor for PM_2.5_ was reported for cigarette smoke detection [16]. 

A micro quartz tuning fork (MQTF) with impact sampling was reported to yield an LOD of 2 µg/m^3^ with PM_2.5_ [17]. Using a 3-D printed virtual impactor (VI) particles smaller than 1.05 µm are separated into a major flow channel and impacted onto a surface acoustic wave (SAW) detector, where a layer of glycerol film served to enhance surface adhesion for capturing them [18]. This way, the PM_1_ fraction of silicon dioxide particles with diameters in the range of 0.1–4 µm was measured. In another approach, a VI was combined with an FBAR for PM_2.5_ detection [19]. In this case instead of impact, thermophoresis (TP) was used for sampling monodisperse polystyrene latex beads. Nevertheless, in these approaches a further separation of the sampled particle fraction into size bins was lacking.

For separating particles of a defined size bin from a polydisperse aerosol, a cascaded arrangement of two impactors (CI) connected in series in the air flow was proposed for PM_2.5_ [20] and PM_0.1_ [21]. For detection, either a thin-film piezoelectric-on-silicon (TPoS) oscillator or a thermal-piezoresistive lateral resonator (TPR) was used, offering fixed bin sizes of 1.03 to 2.54 µm and 40 to 140 nm, respectively. 

Sizing using an impactor or a filter, by which particles of a specific size are separated from the total flow according to their aerodynamic and geometric diameters, respectively, suffers from certain drawbacks. The impactor-based approaches require noisy and power-consumptive pumps for achieving sufficiently high particle velocities and flow rates. Furthermore, to reduce re-bouncing of particles from the mass-detecting resonator body, it should be coated with silicone oil or its surface should be roughened. Separation of particles with a micro filter (µF) in a µFC in the resonator is limited by the surface of µFC wall, which has to be hydrophobic to let particles pass through without excessive wall loss. 

Instead of its inertia as in the case of impactors, differential mobility of charged particles in an electrostatic field is considered in the remainder, which can separate polydisperse particles into several size bins in the diameter range from 50 to 200 nm (Table 1). This way, a more comprehensive characterization of air quality based on portable DMPS instruments was shown to be feasible [22,23]. Remaining drawbacks of the reported designs are supply voltages in the kV range needed for both a charging unit and the DMA, a micro pump for air-flow generation, and a sensitive electrometer for particle detection. Furthermore, these instruments are still rather bulky with DMA channel dimensions of 1 × 5 × 50 mm^3^ [22] and a total weight of 1.0 kg (without battery) and 0.95 kg of standalone miniature UFP sizers, respectively [22,24]. 

## 3. Microfluidic Channel-Based Mobility-Selective Particle Sizer

To face the limitations of current portable DMAs, we developed a single-chip MEMS, which consists of a µFC for conducting a particle-laden air flow and an electrostatic field for mobility-selective sampling of the positive-charged fraction on a PMC for gravimetric sensing, which is described in the following.

### 3.1. Particle Size Separation Using a Differential Mobility Analyzer

A DMA is a size-selective charged particle filter, which separates particles of a given mobility from an aerosol flow depending on particle mobility and a tunable applied voltage. If combined with a particle-concentration detector, it is called a DMPS [ISO 15900].

When force on a charged particle in an electrostatic field equals Stokes drag force, its electrical mobility *Z*_p_ depends on particle radius *d*_p_ according to [28]:

(1) $$Z_{p}=\frac{nqC_{c}}{3\pi \eta d_{p}}$$

with the number of electrical charges per particle n and the Cunningham slip correction factor *C*_c_ which both depend on *d*_p_, the elementary charge *q* = 1.6 × 10^−19^ C, and the dynamic viscosity of air *η* = 1.8205 × 10^−5^ kg/m/s. For NPs of *d*_p_ = 50 nm we can approximate *n* ≈ 1 and *C*_c_ ≈ 5 and calculate a mobility of *Z*_p_ ≈ 10^−7^ Cs/kg.

Conventionally, a DMA is arranged in a coaxial tube-wire configuration, i.e., a wire (of radius *r*_1_) in a channel (of radius *R*), between which an electrostatic voltage *V*_es_ is applied. Along the channel axis, herein, electrically charged particles are carried at a rate *Q* in a laminar air flow and drift towards the wire or the wall, depending on their electrical polarity. Positive-charged particles of diameter *d*_p_ and mobility *Z*_P_, that enter the channel at a distance *r*_2_ from the wire axis, can reach a negative-polarized wire after propagating over the length of the tube *L*_ch_, if a voltage *V*_es_:

(2) $$V_{\mathrm{es}}=\frac{Q\ln\left(\frac{r_{2}}{r_{1}}\right)}{2\pi L_{\mathrm{ch}}Z_{P}}$$

is applied. 

The separation/sampling efficiency *ξ* of particles, which enter the tube with uniform size distribution, is given by the ratio of capture cross section to total area of air flow: The capture cross section is the annular area *π(r*_p_^2^ − *r*_1_^2^) defined by the maximum distance *r*_p_ from the wire, at which a propagating particle is captured, and the wire radius *r*_1_. The total area of particle flow can be approximated by the tube cross-sectional area (π*R*^2^ >> π*r*_1_^2^). We thus yield [28]:

(3) $$\;\xi \left(V_{\mathrm{es}},d_{p}\right)\approx \frac{r_{p}^{2}-r_{1}^{2}}{R^{2}}=\frac{2}{3\eta Q}\frac{L_{\mathrm{ch}}}{\ln\left(\frac{R}{r_{1}}\right)}n\left(d_{p}\right)qC_{c}\left(d_{p}\right)\frac{V_{\mathrm{es}}}{d_{p}}$$

In the size range of *d*_p_ = 20 nm to 300 nm, the parameters n and *C*_c_ depend on *d*_p_ whereby *C*_c_ decreases with *d*_p_ while *n* increases with it. Therefore in total, separation/sampling efficiency will increase with decreasing particle size.

### 3.2. Micro-Fluidic Channel with Integrated Piezoresistive Micro Cantilever

**Figure 2:**
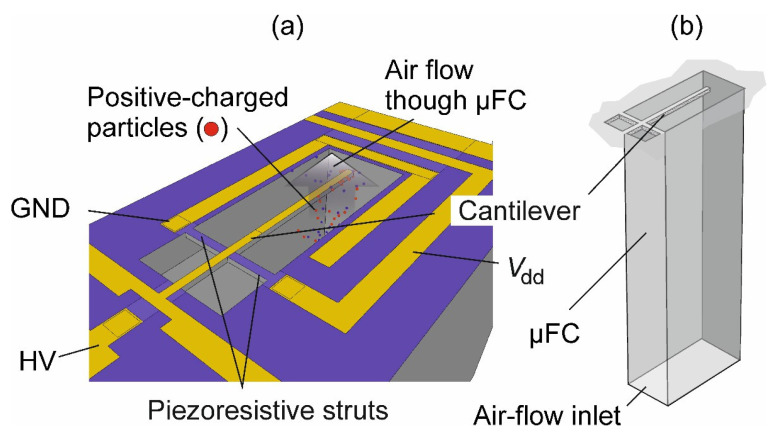
(**a**) Schematic of a micro cantilever with released piezoresistive struts (as strain gauge supplied by *V*_dd_) located in a micro-fluidic channel (µFC) guiding a particle-laden air flow. The cantilever is negatively polarized using a variable direct current (dc)-voltage (HV) with respect to the µFC walls, which are on ground potential (GND). In (**b**) a schematic of the complete µFC is displayed with the air-flow inlet at the bottom of the MEMS die.

Figure 2 shows a schematic of a µFC with an integrated low-mass PMC resonator for electrostatic separation and sampling of NPs. Charged particles sucked-in at the bottom of the µFC and propagating from different lateral positions of its cross-sectional area can be directed towards the small surface area of the cantilever depending on the acting electrostatic drag force. Owing to the small mass of NPs and the constrictions of air flow in the µFC, the device can be operated at low values of separating/sampling voltage (<150 V), flow rate (tens of mL/min) and velocities (tens of mm/s). A small-mass cantilever (16.3 ng) is designed at the top end of the µFC for gravimetric sensing and read-out using a piezoresistive half bridge with resistors represented by released struts extending from the cantilever to the µFC wall. PMC and µFC dimensions are given in Table 2.

In this design the cantilever resonator acts as the particle-sampling electrode, which is arranged perpendicular to the axis of a rectangular µFC guiding a sucked-in aerosol flow. Finite element modelling (FEM) confirmed that the trajectories of positive-charged particles in a laminar air stream are bent towards the negatively polarized cantilever depending on particle mobility (*Z*_p_), air-flow rate (*Q*) and sampling voltage (*V*_es_). 

Fabrication of the PMCs including cantilever, piezoresistive strain gauge, micro-fluidic channel and electrodes for electrostatic particle sampling is based on a novel micromachining process. Its key issue is the underetching of the cantilever without interrupting the cryogenic deep reactive ion etching (cryo DRIE) process. As a consequence, standard bulk silicon wafer material (*n*-doped, 1–10 Ω × cm, <100>) could be used instead of silicon-on-insulator (SOI) wafers, which provides a larger degree of freedom for the design and is less costly. First, thermal diffusion steps are performed for contact formation of the bulk (*n*^+^) as well as doping (*p*) with contact formation (*p*^+^) to the piezoresistive struts. Then, a metallization (gold-chromium) is deposited by e-beam evaporation and patterned using lift-off. Finally, the PMC-µFC is fabricated by sequential front-side and back-side cryo DRIE with SF_6_ and O_2_ as etch gases. Hereby, the front-side process is further divided into two steps: First, anisotropic etching is performed at an O_2_ flow rate of 9 sccm to create vertical cantilever and struts sidewalls. Under these conditions a passivation layer is deposited on the sidewalls which remains stable there at the selected cryogenic temperature. Without interrupting the process, i.e., without warming up the wafer, we then lower the O_2_ flow rate to 4.5 sccm thereby initiating a transition from anisotropy to isotropic etching. After complete release of the cantilever the µFC is then etched from the back side, again under anisotropic etching conditions. Before, photoresist is deposited on the bottom surface of the front-side-etched hole, which then serves as a stop layer for the back-side etching and effectively protects the already released cantilever. Details of the design and fabrication process are available elsewhere [26,27].

**Figure 3:**
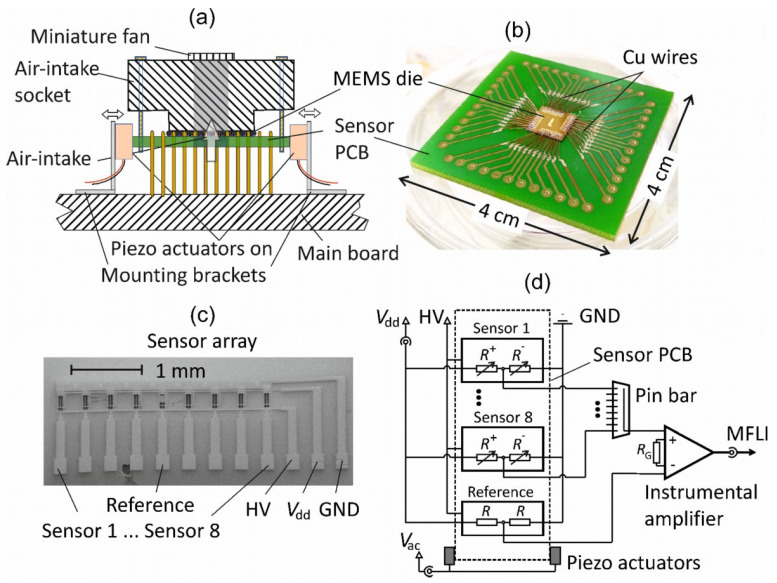
(**a**) Micro electro mechanical system-differential mobility particle sizer (MEMS-DMPS) with sensor printed circuit board (PCB) vertically plugged between the main board and a 3D-printed air-intake socket with a miniature fan (10 × 10 × 2 mm^3^, HY10A03A, SEPA Europe GmbH, Eschbach, Germany), which provides a bottom-to-top airflow in parallel through 4 × 8 µFCs in the MEMS die. The (replaceable) sensor PCB is horizontally pressed in between two miniature multilayer piezo linear actuators (PL055.3, PI Ceramic GmbH, Lederhose, Germany) using mounting brackets connected to the main board. The piezo actuators are operated in differential mode with an excitation voltage *V*_ac_. (**b**) Photograph of the sensor PCB with the MEMS die (8 × 8 mm^2^) glued on a carrier PCB (40 × 40 mm^2^) and connected via soldered Cu wires. (**c**) Scanning electron microscopy (SEM) photograph of an array of 8 PMC-µFCs sensors of identical design, a reference structure and its contact lines to the bridge (*V*_dd_) and sampling voltage (HV) and ground (GND). (**d**) Electric block diagram of the sensor PCB with further read-out components and their interconnections on the main board including an instrumental amplifier (INA217, TI, USA) followed by a Lock-in amplifier (MFLI, Zurich Instruments) and BNC connectors for read-out of the measurement signal, the excitation voltage (*V*_ac_) and *V*_dd_ as well as the HV supply pin and GND.

In Figure 3a a schematic of the MEMS-based differential mobility particle sizer (MEMS-DMPS) is displayed. Here, the MEMS die is mounted upright on a printed circuit board (PCB), which is screwed to a 3D-printed socket with a miniature fan (10 × 10 × 2 mm^3^, HY10A03A, SEPA Europe GmbH, Eschbach, Germany) for intake and flow of air forced through 4 × 8 parallel micro-fluidic channels (µFCs) in the MEMS die. This sensor PCB is vertically plugged on the main board by pressing it between two mounting brackets. Furthermore, two miniature multilayer piezo linear actuators (PL055.3, PI Ceramic GmbH, Lederhose, Germany) are arranged under force-closure between the PCB and the mounting brackets. The piezo actuators are operated in differential mode for resonant excitation of the sensor PCB with a voltage *V*_ac_. The resulting in-plane oscillation of the cantilevers leads to tensile/compressive deformation of the piezoresistive struts (R^+^/R^−^), which is read out via an instrumentational amplifier. Figure 3b shows a photograph of the sensor PCB with the MEMS die (8 × 8 mm^2^) comprising the 4 × 8 arrays of piezoresistive-micro-cantilevers in micro-fluidic-channels (PMC-µFC), which is glued upright onto the sensor PCB (40 × 40 mm^2^). The MEMS die is connected to the PCB via soldered Cu wires. Figure 3c shows a scanning electron microscopy (SEM) photograph of one PMC-µFC array including a reference structure, whose electric block diagram is displayed in Figure 3d. Furthermore, the instrumental amplifier (INA217, TI, USA) followed by a Lock-in amplifier (MFLI, Zurich Instruments, Zurich, Switzerland) are displayed for read-out of the frequency-dependent amplitude and phase signals as well as the supply ports of the excitation voltage (*V*_ac_) of the piezo actuators, the supply voltage of the piezoresistive strain gauge (*V*_dd_) and the HV supply. 

### 3.3. Carbon Aerosol Mass-Concentration Measurement

**Figure 4:**
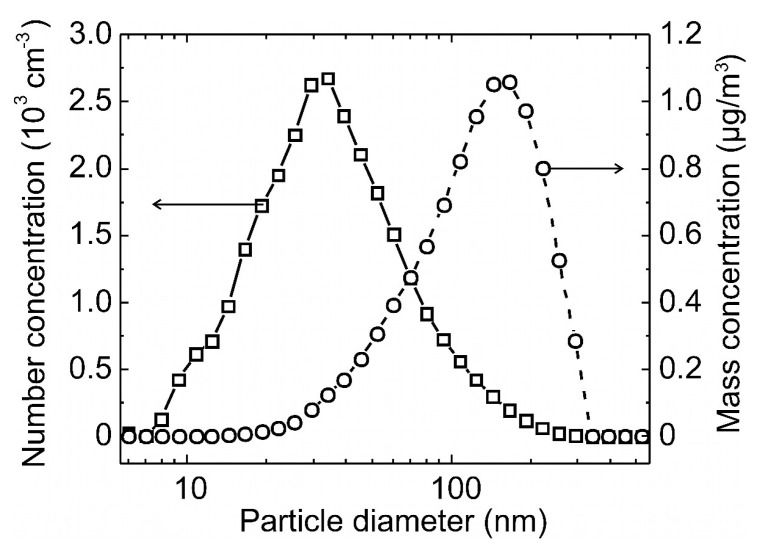
Average size distribution of a nebulized polydisperse carbon aerosol measured with fast mobility particle sizer (FMPS).

For sensor testing, a stable test aerosol was generated using a 6-Jet Nebulizer (BGI Inc., Butler, NJ, USA) with a suspension of carbon NPs (<50 nm, Sigma-Aldrich, Taufkirchen, Germany) in isobutanol and deionized (DI) water. The resulting droplets, dried using a diffusion dryer (TSI Inc., Model 3062, Shoreview, MN, USA), were sprayed into a sealed, temperature- and humidity-controlled chamber (23 °C, 40% relative humidity (RH)) and circulated therein using a ventilation fan. A laboratory fast mobility particle sizer (FMPS) (5.6–560 nm, 32 size bins, TSI Inc., Model 3091, Shoreview, MN, USA) was used as a reference particle sizing instrument. Figure 4 shows a typical mean size distribution (number and mass concentration) of the nebulized polydisperse carbon aerosol of constant mass concentration of 10 µg/m^3^ measured with FMPS over 1 h, which is within the typical range of 4.7 to 19.8 µg/m^3^ of PM_0.1_ pollution to be monitored and controlled in workplace environments [29]. 

The size distribution of average particle number concentration *c*_n_ of the nebulized poly-disperse carbon aerosol in Figure 4 shows a single-size mode with a maximum at *d*_p_ ≈ 30 nm, from which mass concentrations *c*_m_ were calculated assuming spherical shape and uniform density of *ρ*_carbon_ = 2.26 g/cm^3^ of the particles:

(4) $$c_{m}\left(\mathrm{in}\;\frac{\mu g}{m^{3}}\right)=10^{9}\times \frac{\pi }{6}\rho_{\mathrm{carbon}}\left(\mathrm{in}\;\frac{\mathrm{kg}}{m^{3}}\right)\times d_{p}^{3}\left(\mathrm{in}\;{\mathrm{cm}}^{3}\right)\times c_{n}\left(\mathrm{in}\;\frac{\#}{{\mathrm{cm}}^{3}}\right).$$

Here, the maximum of the size distribution has shifted towards a larger diameter of *d*_p_ ≈ 150 nm due to the cubic dependence of particle mass on the diameter (~*d*_p_^3^), which leads to a larger contribution of the bigger particle fraction to *c*_m_ compared to *c*_n_.

Synchronized with the FMPS, one of the PMC-µFCs of the MEMS-DMPS (see Figure 3) was operated in the aerosol chamber. The selected operating parameters are given in Table 3. 

**Figure 5:**
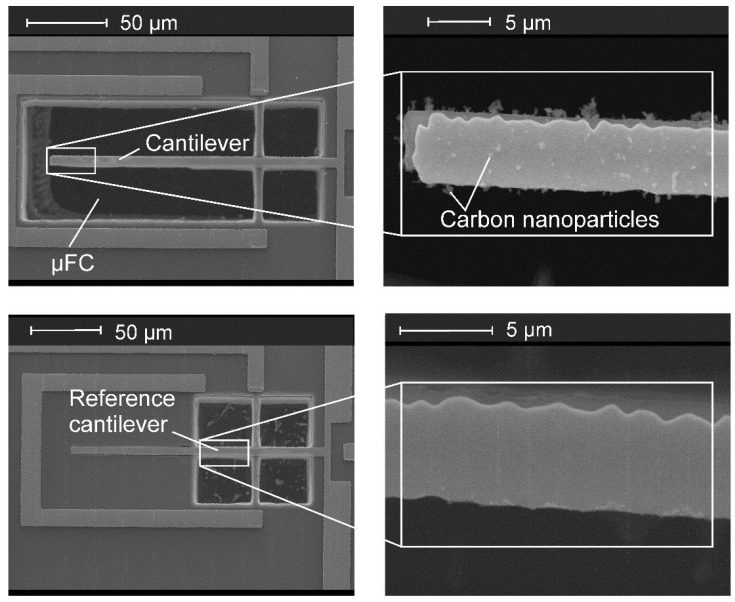
Scanning electron micrograph (SEM) of a PMC-µFC after carbon aerosol particles sampling using *V*_es_ = −30 V at a mass concentration in the chamber of 10 µg/m^3^ for 1 h. Sampled carbon NPs are visible only on the cantilever in a free-etched µFC (**upper**), while the reference structure without µFC does not show any precipitated particle (**lower**).

Figure 5 shows SEM images of a PMC with µFC and a reference structure represented by a not-released cantilever without µFC. Particle separation/sampling and sensing was undertaken simultaneously, i.e., the PMC was connected to the HV supply for setting-up the electrostatic field while resonance frequency of cantilever was measured by frequency sweeps [27]. Carbon NPs captured during separation/sampling are clearly visible in the SEM images of the PMC in Figure 5 taken at the end of the measurement run, whereas they do not appear on the reference structure. 

The measured frequency shift rate at *c*_m_ = 10 µg/m^3^ and *V*_es_ = −30 V amounts to 4.7 ± 0.5 Hz/min. The LOD is 0.75 µg/m^3^. Inherent fluctuations of particle size distribution in small sample volumes may have caused the measured uncertainty of frequency shift rate of ~10%. At our sampling time of *t*_s_ = 9.5 min and an air flow rate of *Q* = 0.3 mL/min, we have a sample volume of vs. ~3 mL, which at the measured concentration of 10 µg/m^3^ corresponds to a total particle mass in the sample of ~30 pg. In agreement with our finding, a 10% uncertainty can be expected at a total sample mass of 30 pg according to model calculations for PM_1_ [30].

## 4. Aerosol Particle Sizing Using the Micro Electro Mechanical System-Differential Mobility Particle Sizer (MEMS-DMPS)

### 4.1. Electrostatic Particle Separation

The PMC-µFC device in Figure 2 can be operated as a DMA for which according to Equation (3), the number of captured particles increases with the sampling voltage and via its size with electrical particle mobility. For confirmation, we performed FEM using COMSOL Multiphysics showing that at sampling/separation voltages of −4 V, −26 V and −140 V positive-charged NPs of diameters of 5 nm, 50 nm and 2.5 µm, respectively, selectively precipitate on the cantilever with maximum efficiencies of nearly 60% to 80% [26]. With its ability to detect sampled particle size bins on a cantilever balance, we can consider this device as a MEMS-DMPS with the resonance-frequency shift rate of the PMC as output signal.

The sampling efficiency *ξ*(*V*_es_, *d*_p_), which is necessary for retrieving the size-distribution of mass concentration of an aerosol from a measured voltage dependence of frequency-shift rate can be determined if the distribution of the number of electrical charges per particle *n* is known [31]. Conventionally, for this purpose aerosol samples with Boltzmann and unipolar charge distributions are prepared. A radioactive source as diffusion neutralizer or a unipolar corona charger are used [32] which, however, are accompanied by a possible excessive mobility overlap between different size bins due to multiple charging in case of unipolar diffusion charging [31]. 

Instead, in this study, we dispense with an additional charging unit and sample only the naturally positive-charged aerosol fraction. As a quantitative measure of the charging state of a naturally charged aerosol we adopt the size and charge distributions measured in the range of *d*_p_ = 20 nm to 400 nm with a NaCl aerosol [33], which we consider as a model system for aerosol charge distributions generated by a nebulizer. We assume that this non-equilibrium charge distribution can describe not only NaCl but also other nebulized aerosols with sufficient accuracy even if, in general, the charge distribution may be dependent on the specific aerosol nebulizer configuration and the chemical composition of the nebulized solution. In the following, we describe a method based on *ξ*(*V*_es_, *d*_p_) determined accordingly (cf. Appendix A and Appendix B), which we propose for retrieving the size-distribution of nebulized aerosols from measurements with the MEMS-DPMS. 

Particle sampling on a cantilever resonator yields a resonance-frequency shift rate of [13,25]:

(5) $$\frac{\Delta f}{\Delta t}\left(V_{\mathrm{es}},d_{p}\right)=\frac{f_{0}}{2m_{0}}\frac{\Delta m}{\Delta t}=\frac{f_{0}}{2m_{0}}\frac{\Delta t}{\Delta V}\frac{\Delta V}{\Delta t}\frac{\Delta m}{\Delta t}=\frac{f_{0}}{2m_{0}}\frac{V_{s}}{t_{s}}\frac{\Delta m}{\Delta V}=\frac{f_{0}}{2m_{0}}Q\xi \left(V_{\mathrm{es}},d_{p}\right)c_{m}$$

with the particle mass concentration *c*_m_, the air flow rate *Q* = *V*_s_/*t*_s_, the sample volume *V*_s_, the sampling time *t*_s_, the resonance frequency *f*_0_ and the resonator mass *m*_0_. The frequency shift rate depends via the separation/sampling efficiency *ξ*(*V*_es_, *d*_p_) on the applied voltage *V*_es_ between the cantilever and the µFC wall as well as on the particle’s diameter *d*_p_ (cf. Equation (3)). Here we consider ranges of particle size and sampling voltage separated in *u* size bins, i.e., *l* = 1, …, *u* and voltage steps, i.e., *k* = 1, …, *v* with *v* ≥ *u*, respectively, define $\xi_{\mathrm{kl}}\left(V_{\mathrm{es},k},d_{p,l}\right)$ as the efficiency for capturing particles of the *l*-th size bin at the *k*-th sampling voltage and replace Equation (5) by a matrix equation:

(6) $$\left(\begin{array}{c} {\left[\Delta f/\Delta t\right]}_{1}\\ \vdots \\ {\left[\Delta f/\Delta t\right]}_{v} \end{array}\right)=\frac{f_{0}}{2m_{0}}Q\left(\begin{array}{ccc} \xi_{11} & \cdots & \xi_{1u}\\ \vdots & \ddots & \vdots \\ \xi_{v1} & \cdots & \xi_{vu} \end{array}\right)\left(\begin{array}{c} c_{m,1}\\ \vdots \\ c_{m,u} \end{array}\right)$$

The vector of mass concentrations (*c*_m,l_) (represented by the *u* size bins of *l* = 1, …, *u*) is multiplied by the efficiency matrix $\xi_{\mathrm{kl}}\left(V_{\mathrm{es},k},d_{p,l}\right)$ and thus is transformed to a vector of frequency-shift rates ([Δ*f*/Δ*t*]_k_) (represented by the *v* voltage values of *k* = 1, …, *v* with *v* ≥ *u*). 

We use FEM to determine $\xi_{\mathrm{kl}}\left(V_{\mathrm{es},k},d_{p,l}\right)$ assuming a uniform distribution of particle size across the µFC cross-section area at the position, where the air flow enters the µFC (cf. Appendix B). For the size range of the polydisperse aerosol we take 20 to 500 nm [23] and separate it into *u* = 6 size bins, which we set according to the 24 bins of a standard FMPS reference instrument as defined in Table A4, and represent them by mean diameters of $d_{p,l}=$ 24.1 nm, 43.2 nm, 77.3 nm, 138.0 nm, 245.9 nm, 437.7 nm for *l* = 1, …, 6. The elements of the 6 × 6 efficiency matrix in Equation (6) are obtained by averaging the respective elements of the 6 × 24 efficiency matrix in Equation (A1):

(7) $$\left(\begin{array}{ccc} \xi_{11} & \cdots & \xi_{16}\\ \vdots & \ddots & \vdots \\ \xi_{61} & \cdots & \xi_{66} \end{array}\right)=\left(\begin{array}{cccccc} 0.021 & 0.028 & 0.033 & 0.025 & 0.021 & 0.015\\ 0.034 & 0.5 & 0.047 & 0.042 & 0.033 & 0.021\\ 0.061 & 0.08 & 0.093 & 0.077 & 0.049 & 0.028\\ 0.1 & 0.161 & 0.175 & 0.147 & 0.099 & 0.052\\ 0.109 & 0.225 & 0.299 & 0.273 & 0.186 & 0.085\\ 0.111 & 0.227 & 0.325 & 0.365 & 0.307 & 0.162 \end{array}\right)$$

As expected from Equation (3) the values of the efficiency matrix in Equation (7) show a trend towards larger values with increasing applied voltage for all size bins (i.e., each column). For each voltage, maximum efficiencies are found for a medium bin size of around 100 nm. The maximum value of *ξ* = 36.5% nearly corresponds to the expected fraction of positive-charged particles of ≈ 40% in a nebulized NaCl aerosol (see Appendix A) indicating that almost all positive-charged particles of *d*_p_ = 100 nm were sampled at the highest *V*_es_. The decrease of efficiency with increasing particle size above 100 nm can be expected according to Equation (3). Due their larger inertia, the trajectories of such larger particles will not be sufficiently bent towards the cantilever to be captured there. The visible efficiency drop for small-size particles reflects the remarkably smaller positive-charged fraction of particles of *d*_p_ < 100 nm and can also be related to particle loss by diffusion or Brownian motion to the channel wall. 

### 4.2. Carbon Particle Sizing Using the MEMS-DMPS

**Figure 6:**
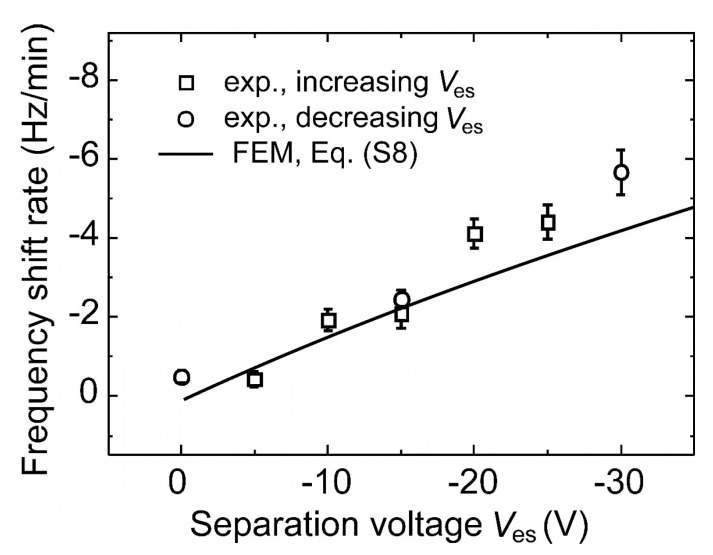
Frequency-shift rate and calculated curve using Equation (A8) from finite element modelling (FEM) measured at different sampling voltages.

We measured the frequency shift rate Δ*f*/Δ*t* of our MEMS-DMPS at sampling voltages *V*_es_ that varied from –30 V to 0 and from 0 to −25 V with carbon UFPs of a constant mass concentration of 10 µg/m^3^ and polydisperse size distribution (see Figure 4). With the MEMS-DMPS, each Δ*f*/Δ*t* value was determined from the frequency-shift measured after sampling times of *t*_s_ = 9.5 min by averaging over 15 times repeated frequency sweeps. In Figure 6 these measured values are compared with $\frac{\Delta f}{\Delta t}\left(V_{\mathrm{es}}\right)$ according to Equation (A8) from FEM. Reasonable agreement is visible for small sampling voltages *V*_es_ ≤ |−15 V|. Above this range, FEM shows a flatter dependence on *V*_es_ than expected from the experiment. As a possible reason for this, differences in charge distribution between the reference aerosol (NaCl) and carbon may be considered. Furthermore, the capture cross-section, i.e., the maximum distance *r*_p_ at which a propagating particle will be captured on the wire, depends on the electrical field around the wire, which is not considered in the modelling. An increase of *r*_p_ can be expected at increasing voltage *V*_es_, which corresponds to a larger cross-section than expected and may lead to the larger frequency-shift rate visible in the experiment. 

**Figure 7:**
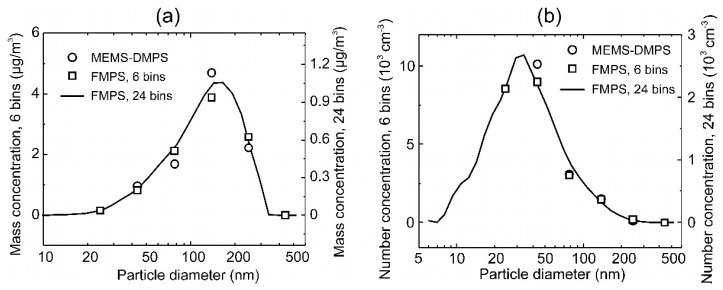
Size distributions of mass (**a**) and number (**b**) concentrations of a nebulized polydisperse carbon aerosol determined for the novel MEMS-DMPS vs. the values measured by FMPS.

**Figure A2:**
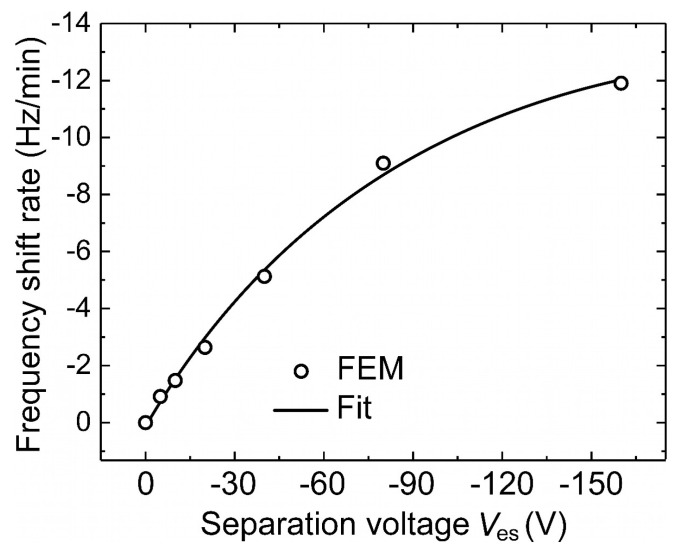
Frequency shift rate determined by FEM for different sampling voltages and fitted curve using Equation (A8).

For retrieving the size distribution of the considered carbon aerosol, Equation (6) was used to transform the given vector of frequency shift rates ([Δ*f*/Δ*t*]_k_) (with *k* = 1, …, *v*) at *V*_es_ = −5 V, −10 V, −15 V, −20 V, −25 V, and −30 V (FEM values in Figure A2) back into a vector of mass concentrations *c*_m,l_ (*l* = 1, …, *u*) at $d_{p,l}=$ 24.1 nm, 43.2 nm, 77.3 nm, 138.0 nm, 245.9 nm, and 437.7 nm. For this, the system of six equations (corresponding to Equation (6)):

(8) $${\left[\frac{\Delta f}{\Delta t}\right]}_{k}=\frac{f_{0}}{2m_{0}}Q\frac{\Delta m}{\Delta t}\sum_{l=1}^{u}\xi_{\mathrm{kl}}c_{m,l};\;k=1,\;\ldots ,\;v$$

was solved using the approximation method of the non-negative least squares in MatLab (*Isqnonneg*()). The results are plotted in Figure 7 superimposed to the size distribution measured using FMPS in the original 24 size bins as well as after compression into the 6 bins of the MEMS-DMPS. The corresponding number concentrations were recalculated from the mass concentrations according to Equation (4). We find good agreement within the entire size range except the smallest bin of $d_{p,1}=$ 24.1 nm, where a zero concentration value was obtained with the FEM data of the MEMS-DMPS. Here, a mass concentration of 0.16 µg/m^3^ (*c*_n,1_ = 8.6 × 10^3^ cm^−3^) was found by FMPS, which is far below the LOD of the MEMS-DMPS of 0.73 µg/m^3^. For the next bin of $d_{p,2}=$ 43.2 nm, where we have a mass concentration of 0.84 µg/m^3^ (*c*_n,2_ = 9.0 × 10^3^ cm^−3^) according to the FMPS, the MEMS-DMPS yields 0.97 µg/m^3^ (*c*_n,2_ = 10.1 × 10^3^ cm^−3^), which is in quite good agreement of 12% (15%).

From the number concentrations, we can calculate the amount of sampled NPs by the MEMS-DMPS for each considered diameter bin from the sucked-in air volume of vs. ≈3 µL at an air flow rate *Q* ≈ 0.3 µL/min and a sampling time *t*_s_ = 9.5 min. We find total particle numbers ranging from ≈ 30,000 particles for the bin of the smallest diameters (*l* = 2, *d*_p_ = 43.2 nm) to 366 particles for the bin of the largest diameters (*l* = 5, *d*_p_ = 249.9 nm), which cause statistical errors ranging from 0.6% to 5.2%, respectively. 

**Figure 8:**
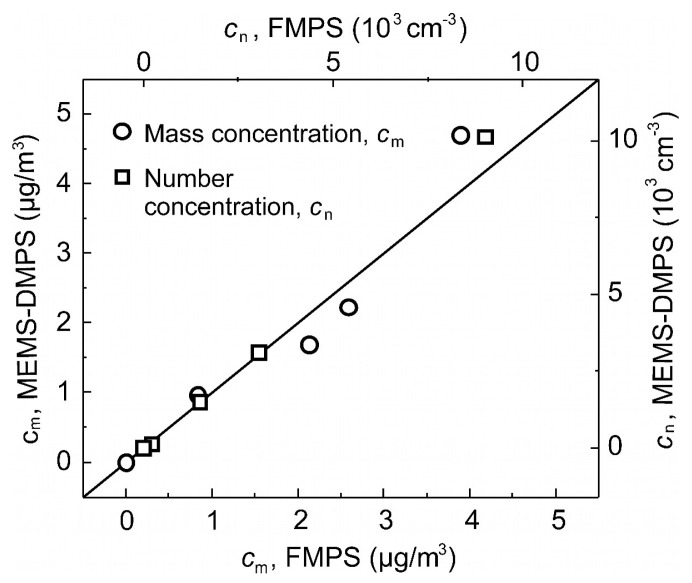
Correlation plot of mass concentration *c*_m_ and number concentration *c*_n_ of a nebulized poly-disperse carbon aerosol determined for the MEMS-DMPS in six size bins vs. the measured FMPS values.

Figure 8 shows a correlation plot of the mass and number concentrations obtained by FEM with the MEMS-DMPS vs. the values measured by FMPS, indicating small maximum deviations of less than 13.6% and 11.1% for mass and number concentration, respectively. 

## 5. Regeneration of the MEMS-DMPS

For continuous use, sampling-based particle monitors/sizers need periodical refreshing by removing the deposited particles from the sampling body or exchanging it for a clean one. In the case of gravimetric detection, the chip containing the resonant device would have to be discarded, which in our case was the MEMS-DMPS chip. To avoid this, cleaning methods of the PMC were proposed and investigated. For removing NPs from a surface, the strong adhesive force acting on the particle has to be surmounted. This can be accomplished with an efficiency of nearly 100% in liquid solvent under the assistance of ultrasonic agitation [34]. Alternatively, a carbon dioxide snow jet can be used to dislodge particles by the impact of solid ice sprayed to the resonator surface under small angle of incidence [35]. Since these methods usually require de-mounting of the sensing chip from its package, it will not be practicable for an unskilled end user. 

Adhesion force increases linearly with diameter, while drag force acting on particles in an air flow is proportional to *d*_p_^2^. Therefore, NPs are much more difficult to remove than large particulates. However, strong adhesion may cause NPs to agglomerate into larger deposits (Figure 5). For daily cleaning, it might thus be sufficient to remove those excessive deposits of large agglomerates by purging clean air through the sampling head, for which the drag force by the air flow can overcome adhesion. In addition, adhesive forces on the NPs can be considerably lowered by a solvent condensed onto the cantilever surface, e.g., from an acetone steam, as shown recently with the PMC of the portable Cantor instrument [25]. 

To check this approach, we investigated a PMC loaded with excessive particulate deposits. We only unplugged the sampling unit from the main board, without de-mounting the sensor PCB from the air-intake socket. Then, we fixed it air-tight on a box containing a reservoir of acetone and switched the fan on to let the acetone steam purge around the PMC and condense there for 10 min. After separating the sensor PCB from the acetone steam, we allowed particles to be removed with the evaporating acetone within a further 5 min in a forced clean-air flow, i.e., with the fan switched on. For confirmation of the refreshing, we re-mounted the sensor PCB to the main board and measured resonance-frequency shift and corresponding mass loss. By repeating this procedure four times, we observed a regeneration of the cantilever indicated by a positive frequency shift rate of 8.7 Hz/min corresponding to a mass removal rate of ≈0.7 pg/min. An extension of the purging time in acetone did not further accelerate particle removal. Thus, about 43 min will be necessary to remove a total of 30 pg deposited mass from a 1 h operation in 10 µg/m^3^ aerosol (see Figure 5). Nevertheless, further experiments, e.g., with other solvents and varied times and flow rates of clean-air purging, will be necessary to improve the refreshing characteristics of cantilevers. 

## 6. Conclusions

A differential mobility ultrafine particle (UFP) sizer was described based on a resonant piezoresistive micro cantilever (PMC) in a micro-fluidic channel (µFC) integrated as a differential mobility particle sizer in a single-chip microelectromechanical system (MEMS-DMPS). Sizing of a carbon nanoparticles aerosol was shown by electrostatic mobility separation of the positive-charged fraction in the µFC and capturing them on the PMC. An inversion method was described based on a priori known charge distribution of the nebulized carbon aerosol combined with finite element modelling (FEM) for retrieving particle size in four bins between ~40 nm and ~250 nm. Mass concentrations in the range of 0–5 µg/cm^3^ showed good agreement of better than 14% of the novel MEMS-DMPS and was found with a simultaneously operated standard reference instrument. The new device offers some advantages vs. electrometer-based micro-fluidic UFP sizers for mobile operation, e.g., an order of magnitude lower separation/sampling voltage and a noiseless miniature fan for air suction. One-chip integration of separating/sampling/detecting components in a standard bulk silicon wafer is a further benefit with respect to reliability and high-volume manufacturing. Polluted cantilevers can be refreshed without de-mounting from a package by purging them alternately in acetone steam and clean air. For a MEMS-based fast mobility particle sizer (MEMS-FMPS) an array of 8 micro channels and cantilever resonators can be operated in parallel at a different fixed sampling voltage for each element in the range of −20 V to −160 V, which will be addressed in a future work. 

## Figures and Tables

**Table 1:** Gravimetric aerosol particle detectors for personal air-quality monitoring. For comparison, three electrometer-based (EM) setups are included offering the capability of particle separation into several size bins. Abbreviations are given below ^1^.

Detection Method	LOD (µg/m^3^)	Sampling Method	Separating Method	No of Bins	Particle Size	Ref.
MEMS-EPC	15	EP	µF/IF	-	PM_2.5_/PM_1_	[13]
	1.4/0.7		-	-	NPs	[14]
	2	µF	µF	-	PM_2.5_	[15]
MEMS-TPO	50	I	-	-	PM_2.5_	[16]
MQTF	2		IF	-	PM_2.5_	[17]
SAW	-		VI	-	PM_1_	[18]
FBAR	-	TP		-	PM_2.5_	[19]
MEMS-TPoS	10	I	2-stage CI	1	1.03 to 2.54 µm	[20]
MEMS-TPR	-			1	40 to 140 nm	[21]
EM	-	EP	DMA	7	50 to 200 nm	[22]
	-	DF		20	50 to 200 nm	[23]
	-	EP and I and µF	DMA and IF	continuous	20 to 400 nm	[24]

^1^ Limit of detection (LOD), particulate matter of aerodynamic diameter of less than 2.5 µm and 1 µm, respectively (PM_2.5_ and PM_1_), micro electro mechanical system (MEMS), electrothermal-piezoresistive cantilever (EPC), thermal-piezoresistive oscillator/resonator (TPO/R), micro quartz tuning fork (MQTF), surface acoustic wave (SAW), film bulk acoustic resonator (FBAR), thin-film piezoelectric-on-silicon (TPoS) oscillator, electrometer (EM), electrophoresis (EP), thermophoresis (TP), micro filter (µF), Impact (I), disc filter (DF), impactor filter (IF), virtual impactor (VI), cascade impactor (CI), differential mobility analyzer (DMA).

**Table 2:** Parameters of the piezoresistive micro cantilever (PMC)-µFC (*l*, *w*, *h*: length, width, height of the PMC; *L*_ch_, *H*_ch_ and *W*_ch_: length, height and width of the µFC).

Micro Cantilever				Micro Channel		
*l* (µm)	*w* (µm)	*h* (µm)	*m*_0_ (ng)	*L*_ch_ (µm)	*H*_ch_ (µm)	*W*_ch_ (µm)
171 ± 1	10.5 ± 0.4	3.0 ± 0.5	16.3 ± 2.9	270 ± 15	191 ± 1	72.4 ± 0.5

**Table 3:** Operating parameters of the PMC-µFC with carbon NPs (*c*_m_: particle mass concentration; *Q*: air-flow rate; *f*_0_: cantilever resonance frequency; *m*_0_: cantilever mass; *t*_s_: sampling time).

*c*_m_ (µg/m^3^)	*Q* (µL/min); (10^−9^ m^3^/s)	*f*_0_ (kHz)	*V*_es_ (V)	*t_s_* (min)
10 ± 1	306 ± 30; 5.1 ± 0.5	400 ± 5	−5 to −30	9.5

**Figure A1:**
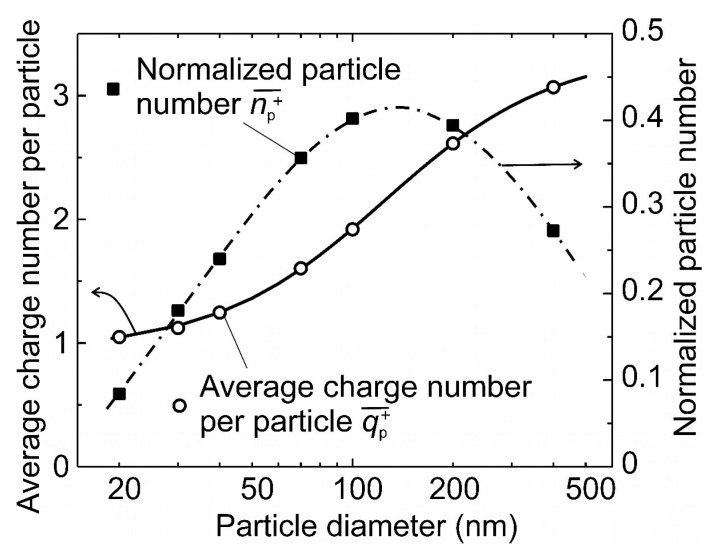
Average charge number per (positive-charged) particle and normalized (positive-charged) particle number of a nebulized NaCl aerosol.

## Data Availability

Not applicable.

## Appendix A. Positive-Charged Fraction of Nebulized Aerosol

Measured fractions $n_{p}^{+}\left(d_{p},n\right)$ of *n*-times (*n* = 1, 2, 3, 4, 5, 6) positive-charged particles with diameters of *d*_p_ = 20 nm, 30 nm, 70 nm, 100 nm, 200 nm, and 400 nm are given in reference [33] for a NaCl aerosol generated by a nebuliser (Table A1). Values for *n* = 5, 6 are determined by extrapolation.

**Table A1:** Fraction $n_{p}^{+}\left(d_{p}\right)$ of *n*-times positive-charged particles of a nebulized NaCl aerosol (data of Simones et al. [33], Figure 4) for particle diameters *d*_p_ (values for *n* = 5 und *n* = 6 were estimated by extrapolation of the charge distribution).

*n*	$n_{p}^{+}\left(d_{p},n\right)$						
	*d*_p_ (nm) = 20	30	40	70	100	200	400
1	0.08	0.16	0.19	0.21	0.185	0.12	0.05
2	0.004	0.019	0.042	0.097	0.12	0.092	0.06
3		0.0014	0.0066	0.034	0.056	0.076	0.06
4			0.0013	0.012	0.027	0.052	0.048
5				0.0035	0.014	0.0353	0.033
6						0.0189	0.0214

From this we then calculate the average number of elementary charges of the positive-charged particle fraction for each particle diameter:

(A1) $$\bar{q_{p}^{+}}\left(d_{p}\right)=\frac{\sum_{n=1}^{6}n\times n_{p}^{+}\left(d_{p},n\right)}{\bar{n_{p}^{+}}\left(d_{p},n\right)}$$

with the corresponding normalized number of positive-charged particles (positive-charged fraction of the particles of the aerosol):

(A2) $$\bar{n_{p}^{+}}\left(d_{p}\right)=\overset{6}{\underset{n=1}{\sum }}n_{p}^{+}\left(d_{p},n\right)$$

The values of $\bar{n_{p}^{+}}$ and $\bar{q_{p}^{+}}$ for particle diameters $d_{p}$ from 20 nm to 400 nm are given in Table A2.

**Table A2:** From the values in Table A1 calculated average normalized fraction of positive-charged particles $\bar{n_{p}^{+}}\left(d_{p}\right)$ and their average charge number $\bar{q_{p}^{+}}\left(d_{p}\right)$.

*d*_p_ (nm)	20	30	40	70	100	200	400
$\bar{n_{p}^{+}}$	0.084	0.1804	0.2399	0.3565	0.402	0.3942	0.2724
$\bar{q_{p}^{+}}$	1.04	1.12	1.25	1.6	1.92	2.61	3.07

In Figure A1 the values of $\bar{n_{p}^{+}}$ and $\bar{q_{p}^{+}}$ given in Table A2 are plotted vs. $d_{p}$ and superimposed with curves respectively fitted using a polynomial function:

(A3) $$y_{1}\left(d_{p}\right)=\frac{a_{1}d_{p}^{2}+a_{2}d_{p}+a_{3}}{d_{p}^{2}+b_{1}d_{p}+b_{2}}$$

We find average charge numbers between about 1 and 3 and positive-charged fraction between about 0.1 and 0.4. The fitting parameters *a*_1_, *a*_2_, *a*_3_, *b*_1_ and *b*_2_ are shown in Table A3.

**Table A3:** Fitted values using Equation (A3) for the average normalized fraction (open squares, broken line) and for the average charge number (open circles, full line), respectively, of the positive-charged particles shown in Figure A1.

$y_{1}$	*a*_1_ (nm^−2^)	*a*_2_ (nm^−1^)	*a*_3_	*b*_1_ (nm^−1^)	*b*_1_
$\bar{q_{p}^{+}}$	−0.281	355.2	−4546	398.4	20,350
$\bar{n_{p}^{+}}$	3.402	92.28	13690	47.97	14,790

## Appendix B. Electrostatic Particle Separation/Sampling Using a MEMS-DMPS

Here we calculate the expected frequency-shift rate of a MEMS-DMPS depending on the applied sampling voltage. We follow the common practice and investigate air-fluidic components for separation of particles in flow channels by computational fluid dynamics (CFD) modelling, e.g., using COMSOL Multiphysics [18,19]. Its electrostatic and laminar flow modules are appropriate for electrical-field and air-flow modelling and the “particle tracing for fluid flow module” for computing particle motion under electrical and air-flow fields, where electrical, drag, gravity, and Brownian forces are embedded [32].

We assume smooth and spherical particles carried by a fluid (air) showing no inter-particle interactions (collisions) and having a uniform distribution across the inlet of the micro channel (µFC). Wall deposition is considered, which may be more probable for small particles due to strong Brownian motion and low particle velocity corresponding to long particle residence in the µFC and can amount up to ≈25% of the total number of nanoparticles (10 nm) at the outlet a µFC (*L*_C_ = 12 µm, *H*_C_ = 0.6 µm, *L*_C_/*H*_C_ = 20) [36]. However, in our case the µFC has a much lower length to width ratio of *L*_C_/*H*_C_ = 3.73, and a correspondingly much lower wall deposition efficiency of ≈5% may be expected.

We then calculate the frequency-shift rate vs. sampling voltage Δ*f*/Δ*t* = f(*V*_es_) with a geometrical model of our single-chip MEMS-DMPS comprising the µFC and the cantilever, which was previously described in detail [27]. We use a number of 1000 × $\bar{n_{p}^{+}}\left(d_{p}\right)$ particles with average charge numbers $\bar{q_{p}^{+}}\left(d_{p}\right)$ and set *d*_p_ corresponding to the average values of *j* = 1, …, 24 size bins of the FMPS from 19.1 nm to 523.3 nm in Table A4. Here, the bins from 6.04 nm to 16.3 nm of the FMPS were not considered, since the expected mass concentrations were below the LOD of the MEMS-DMPS of 0.73 µg/m^3^ [27].

Using FEM, the number of particles $\bar{\Delta n_{p,j}^{+}}$ of the *j*-th size bin with average particle diameter $d_{p,j}$ (*j* = 1, …, 24) is determined which are sampled from the initial ensemble of 1000 × $\bar{n_{p,j}^{+}}$ at *V*_es,i_ (*i* = 1, …, 6), whereby $\bar{n_{p,j}^{+}}$ is the normalized count of positive-charged particles with an average total charge number $\bar{q_{p,j}^{+}}$ (see Appendix A). For particle separation/sampling we select voltages of *V*_es,i_ = –5 V, –10 V, –20 V, –40 V, –80 V, –160 V (*i* = 1, …, 6).

**Table A4:** Numbering of size bins of FEM/FMPS and MEMS-DMPS (*j*, *l*: Number of particle size bin, *d*_p,I,k_: average particle diameter in bin no. *j*, *l*).

FEM/FMPS		MEMS-DMPS	
*j*	*d*_p,j_ (nm)	*l*	*d*_p,l_ (nm)
1	19.1	1	24.1
2	22.1		
3	25.5		
4	29.4		
5	34.0	2	43.2
6	39.2		
7	45.3		
8	52.3		
9	60.4	3	77.3
10	69.8		
11	80.6		
12	93.1		
13	107.5	4	138.0
14	124.1		
15	143.3		
16	165.5		
17	191.1	5	245.9
18	220.7		
19	254.8		
20	294,3		
21	339.8	6	437.7
22	392.4		
23	453.2		
24	523.3		

The resulting sampling efficiencies $\xi_{\mathrm{ij}}\left(V_{\mathrm{es},i},d_{p,j}\right)$ at each of the 6 voltage values (*V*_es,i_) for each of the 24 size bins (*d*_p,j_) can be written as a 6 × 24 matrix:

(A4) $$\left(\begin{array}{ccc} \xi_{11} & \cdots & \xi_{1\;24}\\ \vdots & \ddots & \vdots \\ \xi_{61} & \cdots & \xi_{6\;24} \end{array}\right)$$

Using this efficiency matrix we can now transform the vector of particle number concentration ($c_{n,j}$) (*j* = 1, …, 24) given by the measured size distribution (see Figure 4) into a vector of the number of positive-charged particles $\left(\Delta n_{p,i}^{+}\right)$ (*i* = 1, …, 6) sampled on the cantilever:

(A5) $$\left(\begin{array}{c} \Delta n_{p,1}^{+}\\ \vdots \\ \Delta n_{p,6}^{+} \end{array}\right)=10^{3}\left(\begin{array}{ccc} \xi_{11} & \cdots & \xi_{1\;24}\\ \vdots & \ddots & \vdots \\ \xi_{61} & \cdots & \xi_{6\;24} \end{array}\right)\left(\begin{array}{c} c_{n,1}\\ \vdots \\ c_{n,24} \end{array}\right)$$

Assuming spherical shape of the model particles and uniform density (*ρ*_carbon_ = 2.26 g/cm^3^) the sampled particle mass vector $\left(\Delta m_{i}\right)$ can be calculated from $\left(\Delta n_{p,i}^{+}\right)$ (*i* = 1, …, 6):

(A6) $$\left(\begin{array}{c} \Delta m_{1}\\ \vdots \\ \Delta m_{6} \end{array}\right)=\frac{\pi }{6}d_{j}^{3}\left(\begin{array}{c} \Delta n_{p,1}^{+}\\ \vdots \\ \Delta n_{p,6}^{+} \end{array}\right)$$

and then the related vector of frequency shift rate $\left({\left[\frac{\Delta f}{\Delta t}\right]}_{i}\right)$ (*i* = 1, …, 6) is:

(A7) $$\left(\begin{array}{c} {\left[\frac{\Delta f}{\Delta t}\right]}_{1}\\ \vdots \\ {\left[\frac{\Delta f}{\Delta t}\right]}_{6} \end{array}\right)=\frac{f_{0}}{2m_{0}\Delta t}\left(\begin{array}{c} \Delta m_{1}\\ \vdots \\ \Delta m_{6} \end{array}\right)$$

which are calculated using the values for *f*_0_, *m*_0_ and Δ*t* given in Table 3. In Table A5, the components of the resulting frequency-shift-rate vector are given in dependence of the sampling voltage.

**Table A5:** Numbering and values of sampling voltage for determination of sampling efficiency of the MEMS-DMPS using FEM and for experimental particle sizing using the inversion method.

*i*	*V*_es,i_ (V)	${\left[\frac{\Delta f}{\Delta t}\right]}_{i}\left(\frac{Hz}{min}\right)$
1	−5	0.92
2	−10	1.48
3	−20	2.64
4	−40	5.12
5	−80	9.10
6	−160	11.90

These values are plotted in Figure A2 and then fitted using an analytical formula:

(A8) $$\frac{\Delta f}{\Delta t}\left(V_{\mathrm{es}}\right)=a+b\exp\left(cV_{\mathrm{es}}\right)$$

with coefficients of *a* = 14.1 Hz/min, *b* = −14.2 Hz/min, and *c* = −0.012 V^−1^.
